# Characteristics and Traceability Analysis of Microbial Assemblage in Fine Particulate Matter from a Pig House

**DOI:** 10.3390/ani13061058

**Published:** 2023-03-15

**Authors:** Miao Wang, Siyi Peng, Dongru Liu, Dingbiao Long, Zuohua Liu, Shihua Pu

**Affiliations:** 1 College of Animal Science and Technology, Southwest University, Chongqing 402460, China; 2 Chongqing Academy of Animal Sciences, Changlong Avenue, Chongqing 402460, China; 3 National Center of Technology Innovation for Pigs, Chongqing 402460, China; 4 Scientific Observation and Experiment Station of Livestock Equipment Engineering in Southwest, Ministry of Agriculture and Rural Affairs, Chongqing 402460, China

**Keywords:** particular matter, microorganism, network analysis, microbial traceability

## Abstract

**Simple Summary:**

Fine Particulate Matter (PM_2.5_) can severely harm human and animal health because it can carry many harmful microorganisms and enter the deep respiratory tract. Due to the high breeding density and poor ventilation in large-scale pig farms, the concentration of PM_2.5_ is higher indoors than outdoors. Therefore, it is very important to understand the composition of harmful microorganisms carried by PM_2.5_ in pig houses and trace their sources and interactions with the environment. This study first monitored the environment of a piggery, identified the species and abundance of pathogenic bacteria and allergens on the collected PM_2.5_ samples using high-throughput sequencing, then analyzed the interactions between microbial communities and between communities and environmental factors using network analysis, and finally, used the SourceTracker tool to predict the microbial traceability of PM_2.5_. The results showed that the contribution of feces to producing airborne microorganisms was much higher than that of feed.

**Abstract:**

Fine particulate matter (PM_2.5_) can carry numerous substances and penetrate deep into the respiratory tract due to its small particle size; associated harmful microorganisms are suspected to increase health risks for humans and animals. To find out the microbial compositions of PM_2.5_ in piggeries, their interaction and traceability, we collected PM_2.5_ samples from a piggery while continuously monitoring the environmental indicators. We also identified pathogenic bacteria and allergens in the samples using high-throughput sequencing technology. We analyzed the microbial differences of PM_2.5_ samples at different heights and during different times of day and investigated the microbial dynamics among the PM_2.5_ samples. To better understand the interaction between microorganisms and environmental factors among different microbial communities, we applied the network analysis method to identify the correlation among various variables. Finally, SourceTracker, a commonly used microbial traceability tool, was used to predict the source of airborne microorganisms in the pig house. We identified 14 potential pathogenic bacteria and 5 allergens from PM_2.5_ in the pig houses, of which *Acinetobacter* was the dominant bacterium in all samples (relative abundance > 1%), which warrants attention. We found that bacteria and fungi directly affected the the microbial community. The bacterial community mainly played a positive role in the microbial community. Environmental variables mainly indirectly and positively affected microbial abundance. In the SourceTracker analysis using fecal matter and feed as sources and PM_2.5_ sample as sink, we found that fecal matter made the greatest contribution to both bacterial and fungal components of PM_2.5_. Our findings provide important insights into the potential risks of pathogens in PM_2.5_ to human and animal health and their main sources.

## 1. Introduction

Intensive pig production leads to high density concentrations of particulate matter (PM) in pig houses, resulting in a high incidence of respiratory diseases in pigs and posting serious health risks to pig farm workers [1,2]. Fine particulate matter (PM_2.5_) with a small particle size and a large specific surface area, can easily adsorb and carry harmful substances, and enter the alveoli [3,4]. The threat of PM_2.5_ to public health has attracted worldwide attention [5]. As one of the primary agricultural sources, PM_2.5_ in pig farms has a high concentration and complex components, making it a major threat to pig welfare and the health of pig farm workers.

PM_2.5_ in piggeries is significantly different from that in the atmospheric environment, with a high proportion of microorganisms originating from biological sources. Previous studies have reported high concentrations of airborne microorganisms and their derivatives in farms, including potential pathogenic bacteria, allergens and endotoxins [6]. These substances are harmful to humans and pigs, indicating that the threat of PM_2.5_ to public health in farms may be aggravated by microbial components. In recent years, the rapid development of high-throughput technology has enabled many researchers to explore the changes in microbial composition in the environment by species and their abundance, through DNA sequencing [7,8]. It has been recognized that the abundance and composition of airborne microorganisms are affected by many factors, including environmental variables (temperature, relative humidity, ventilation, etc.), sources, seasonal changes, and human or animal activities, etc. [9,10]. For example, a relatively high temperature is the primary condition for bacterial growth, while low temperature is a limiting factor [11]. It should be emphasized that differences in the structure of airborne microbial communities may alter the proportion and activity of toxicogenic components within them, with consequent changes in the impact on animal and human health [12]. Therefore, a thorough understanding of the relationship between environmental factors and airborne microorganisms is of great importance in assessing their health risks and developing scientific control strategies.

Previous studies have focused on the relationship between airborne microorganisms and the environment in farms mainly based on seasonal differences [13,14]. However, the density, number and growth stage of animals in breeding houses can vary significantly over such a long period, potentially affecting analysis results. Therefore, a quarterly monitoring analysis is also worth exploring. Additionally, although some previous studies have identified PM_2.5_ sources in farms, such as feed, dander, feces, urine, bedding, and skin, the contribution of each source remains unclear [15]. Therefore, paying attention to the dynamic changes of airborne microorganisms in pig houses in the short term and quantitatively analyzing their sources may play a positive role in improving the environment of pig houses.

In this study, environmental parameters in the pig house were monitored in the spring season, while different PM_2.5_ samples were collected at different heights and times of the day. Through DNA sequencing techniques, the potential pathogenic bacteria and allergens were identified, and the interactions between bacterial, fungal and environmental variables were explored by network analysis. On this basis, the source of bacteria and fungi was analyzed using the SourceTracker method. Our findings can provide reference for the environmental regulation of farms.

## 2. Materials and Methods

### 2.1. Pig House Management and Environmental Information Collection

In this study, a fattening pig house in Rongchang District of Chongqing City was selected for environmental monitoring and analysis. The pig house had dimensions of 37.35 × 11.66 × 3.30 m (length × width × height), with each fence measuring 4.15 × 5.24 m long and featuring a leaky floor (4.15 × 1.18 m). In the pig house, a total of 110 pigs, weighing 70 to 90 kg each, were evenly distributed in 12 pig pens. Additional information on the structure, ventilation system, feeding and management of the pig house can be found in Pu et al. [16]. From 10 April to 25 April 2022, environmental data were monitored and samples of indoor environment were collected. The breeders feed pigs at 8:30 a.m. and 3:00 p.m. every day and cleaned the house. Temperature and relative humidity were monitored every 5 min using HOBO (U23-001, Onset, Bourne, MA, USA) and recorded. The PM_2.5_ concentration was measured using the ‘weighing method’ during the day (7:30–19:30) and night (19:30–7:30). The monitoring points for the above indicators were located at 1.7 m and 0.6 m in the middle of the piggery channel. In addition, the ventilation rate in the pig house was automatically recorded after being adjusted by the animal husbandry building ventilation environment controller (TC1208, Chongqing Metea Electronic Technology Co., Ltd., Chongqing, China).

### 2.2. Sample Collection and Processing

PM_2.5_ samples were collected using a quartz fiber filter (MK360, 90 mm) and an environmental particle collector (2030 medium flow intelligent TSP sampler, Qingdao Laoying Environmental Technology Co., Ltd., Qingdao, China). The sampler was placed at a height of 1.7 m and 0.6 m in the middle of the aisle in the pig house. The flow rate of the collector was set to 100 L·min^−1^ for 12 h of continuous sampling. Pretreated filter membranes were placed in the cutting head of the collector and were replaced twice a day at 7–8 am and 19:00–20:00 pm to obtain PM_2.5_ samples corresponding to daytime and nighttime. Feed (Chongqing Huiguang Company, Chongqing, China, 930 model) and fresh pig feces were also collected every other day when the filter membrane was replaced. The collected samples of PM_2.5_, feed and feces were immediately placed in an ice box and sent to the laboratory −80 °C freezer as soon as possible for subsequent analysis.

The collected quartz membrane samples were divided into four categories based on altitude and time of day (High altitude during the day, Low altitude during the day, High altitude at night and Low altitude at night). Ten membranes were randomly selected from each category and processed together using the following procedure. First, the membranes were cut into 1 cm^2^ pieces and placed in a sterilized 250 mL beaker. Then, 150 mL of high purity water (18.2 MΩ) was added. The beaker was sealed with a sterile membrane and placed in a sterile beaker for ultrasonic oscillation for 60 min. Next, the liquid was filtered by using a 0.2 μm membrane (width 50 mm, Tianjin Jinteng Experimental Equipment Co., Ltd., Tianjin, China). The filtered liquid was analyzed for ammonium ion (NH_4_^+^) and nitrate ion (NO_3_^−^) content in the solution by a spectrophotometry-based hash instrument (DR6000, Hach, Loveland, CO, USA). The filtered microporous membranes were collected for subsequent microbial sequencing.

### 2.3. DNA Extraction, High-Throughput Sequencing and Data Processing

The collected PM_2.5_ samples were grouped and mixed based on height and time of day (i.e., day and night); thus, four types of PM_2.5_ samples were obtained. Bacterial and fungal component analysis was performed on each sample, which was repeated three times. At the same time, feed and feces samples were also analyzed for microbial composition for traceability comparison. The names of each corresponding sample are shown in Table 1.

First, the total DNA in each sample was extracted by using Biomarker Soil Genomic DNA Kit (RK02005). Then, the bacterial and fungal genes of the samples were amplified in the following two ways. Bacteria primer information was: F: ACTCCTACGGGAGGCAGCA and R: GGACTACHVGGGTWTCTAAT. The PCR amplification program was 95 °C for 5 min, then 30 cycles of 95 °C for 30 s, 50 °C for 30 s, 72 °C for 60 s, and finally extended at 72 °C for 5 min. Fungal primer information was: F: CTTGGTCATTTAGAGGAAGTAA; R:GCTGCGTTCTTCATCGATGC. The PCR amplification program was run at 95 °C for 5 min, followed by 35 cycles of 95 °C, 50 °C and 72 °C (all for 1 min, and finally extended at 72 °C for 7 min).

The amplified products were purified, quantified and homogenized to creat a sequencing library. Firstly, the quality control of the constructed library was carried out, and the qualified library was sequenced on the Illumina HiSeq 2500 platform. After base call analysis, the original image data file was converted into the original sequencing readings, and the results were stored in the FASTQ file format, containing sequence information (Reads) and corresponding sequencing quality information. Sequence processing includes several steps, including using Trimmomatic v0.33 software to filter the quality of the original readings obtained by sequencing, and then using Cutadapt 1.9.1 software to identify and remove primer sequences to obtain clean readings without primer sequences. Then, the paired sequences were spliced by Usearch v10 software, the clean readings of each sample were spliced by overlap, and the spliced data were filtered according to the length range of different regions. Finally, the chimeric sequence was removed by UCHIME v4.2 software to obtain the final valid data.

### 2.4. Sequence Processing and Bioinformatics Analysis

QIIME software was used to process 16S and ITS1 gene sequences. After low-quality sequences and chimeras were filtered, the effective sequences of bacteria and fungi were normalized to compare the same sequencing depth of all PM_2.5_ samples. Usearch software was used to cluster Reads at a similarity level of 97.0% to obtain operational taxonomic units (OTUs). The Silva database was used as a reference database to use the naive Bayesian classifier combined with the alignment method to perform taxonomic annotations on the feature sequence to obtain the species classification information corresponding to each feature. Sample community composition at each species classification level was then counted. The ACE index was used to measure species abundance, and Shannon index was used to measure species diversity. Principal coordinate analysis (PCoA) was used to analyze the differences among different types of samples. Analysis of similarities (Anosim) was further used to test for differences in beta diversity. Biomarkers of the microbial taxa were analyzed using the linear discriminant analysis (LDA) effect size (LEfSe) analysis with a threshold of 4 for LDA. Network analysis was used to explore the interactions among bacteria, fungi, and environment. Firstly, the Spearman correlation analysis of environmental parameters, bacteria and fungi within and between groups was performed using R language (4.2.1 edition) to obtain the correlation matrix. The *p* value was corrected for the false positive rate. The correlation matrices with correlation coefficients r ≥ 0.7 and r ≤ −0.7 at the *p* < 0.01 level are defined as strong positive and negative interactions, respectively. Gephi software (version 0.9) was used to visualize the interaction among bacterial, fungal, and environmental parameters, and to obtain network topology properties.

### 2.5. Microbial Traceability

This study employed SourceTracker to analyze the microbial composition of PM_2.5_. As a classic microbial traceability tool based on Bayesian algorithm, it has been widely used in medical and health care, soil environment and other fields [17,18]. Here, bacteria and fungi from PM_2.5_ samples were considered as “sinks” for traceability, while bacteria and fungi from feed and fecal samples were considered as ‘sources’. Categorical units that cannot be mapped to the input source were classified as ‘unknown’. In the R language (version 4.2.1), the Source Tracker operating parameters were sparse depth 1000, pre-simulation run 100, restart 10, alpha 0.001, beta 0.01, with high sensitivity, specificity and accuracy.

### 2.6. Statistical Analysis

Conventional statistical data were analyzed using the social science statistical software package (SPSS 26.0, Chicago, IL, USA). GraphPad Prism Version 9 software (GraphPad, San Diego, CA, USA) was used for mapping. For normally distributed data, we performed the unpaired t test, while for non-normal distributions, we used the non-parametric test (Mann–Whitney U test). Data were expressed as mean ± standard error (SD). *p* value less than 0.05 was considered significant. The Relative Standard Deviation (RSD) was obtained by calculating the ratio of the standard deviation to the average predicted value. The purpose of using RSD value was to determine the consistency of the results when applying the default SourceTracker settings, as the variability of SourceTracker when running the model can raise concerns about the accuracy of the prediction results.

## 3. Results

### 3.1. Environment Variables

The environmental variables in the pig house have obvious diurnal variation characteristics (Table 2). Specifically, PM_2.5_ concentration, ventilation rate and temperature were significantly higher during the day than at night (*p* < 0.05). The concentration of PM_2.5_ fluctuated from 22.91 to 95.61 μg·m^−3^. Both by day and at night, the concentration of PM_2.5_ were significantly higher at a height of 1.7 m than that at 0.6 m (*p* < 0.05). The ventilation rate ranged from 6.4 to 47 m³·h^−1^, and low ventilation was maintained at night. Temperature and humidity fluctuated between 12.31–25.18 and 40.06–88.43%, respectively. The humidity was significantly higher at night than during the day (*p* < 0.05). In addition, the contents of water-soluble ions NH_4_^+^ and NO_3_^−^ in PM_2.5_ were 2.07–5.14 μg∙m^−3^ and 0.61–1.31 μg∙m^−3^, respectively. Their concentrations in daytime samples were significantly higher than those at night (*p* < 0.05).

### 3.2. Bacterial and Fungal Community Diversity

Based on the Good’s coverage value (>97%), the sequences generated by Illumina sequencing captured most of the bacteria and fungi in the feed, PM_2.5_, and feces samples. While some differences in microbial diversity were observed among samples, abundance (ACE) and diversity (Shannon) of bacteria were significantly higher than those of fungi (Table 3).

**Figure 1:**
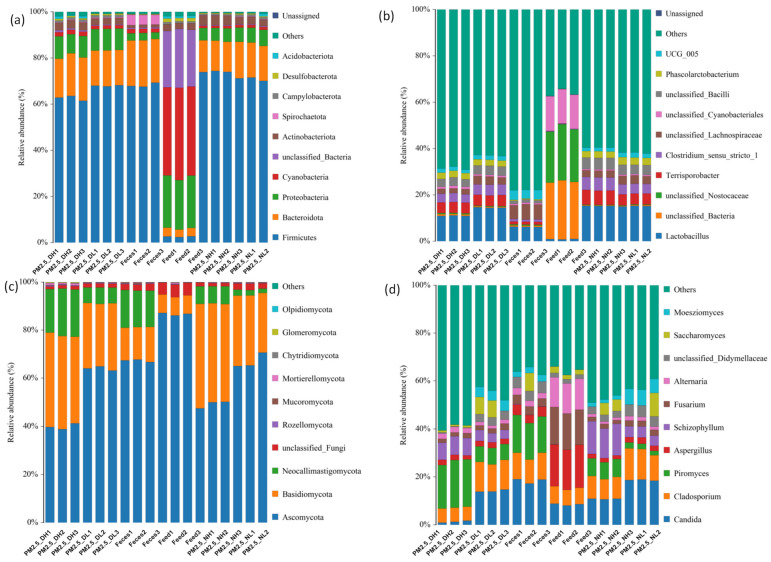
Distribution of bacteria (**a**,**b**) and fungi (**c**,**d**) in samples; (**a**,**c**) indicate composition at the phylum level and (**b**,**d**) indicate composition at the genus level.

At the phylum level, feces and PM_2.5_ samples had similar bacterial compositions (Figure 1a), mainly composed of *Firmicutes* (61.45–74.41%), followed by *Bacteroidota* (13.07–18.44%) and *Proteobacteria* (5.32–9.73%). The bacterial composition of the feed was very different from PM_2.5_ at the phylum level. It was mainly composed of *Cyanobacteria* (38.18–39.97%), followed by *Proteobacteria* (21.47–22.69%). The relative abundance of *Firmicutes* and *Bacteroidota* in feed bacteria were less than 5 %. In terms of fungal composition (Figure 1c), *Ascomycota* (38.73–87.16%) had the highest relative abundance in all samples, followed by *Basidiomycota* (7.64–43.37%). *Neocallimastigomycota* accounted for 1.83–19.73% in PM_2.5_ and feces samples, but was not detected in feed.

At the genus level, *Lactobacillus* had the highest relative abundance of bacteria in PM_2.5_ and feces samples (6.09–15.25%), while it was less than 1% in feed (Figure 1b). In terms of fungi, although the top ten abundances of fungi were consistent among different groups of PM_2.5_, there were some differences in relative abundance (Figure 1d). *Candida* accounted for only 0.94–1.25% of PM_2.5_ samples collected at 1.7 m during the day, while the relative abundance of *Candida* in other PM_2.5_ samples ranged from 10.88% to 19.04%. *Piromyces* (1.83–19.84%) and *Schizophyllum* (3.7–13.61%) also varied greatly among PM_2.5_ samples. *Schizophyllum* was detected in feces, and other fungus of which were close to those in PM_2.5_. The main bacteria in the feed were *Fusarium* (14.67–15.61%), *Aspergillus* (16.72–17.87%) and *Alternaria* (12.31–12.75%), containing some *Candida* (8.04–8.85%) and *Cladosporium* (6.54–7.24%).

### 3.3. Microbial Dynamics in PM_2.5_

**Figure 2:**
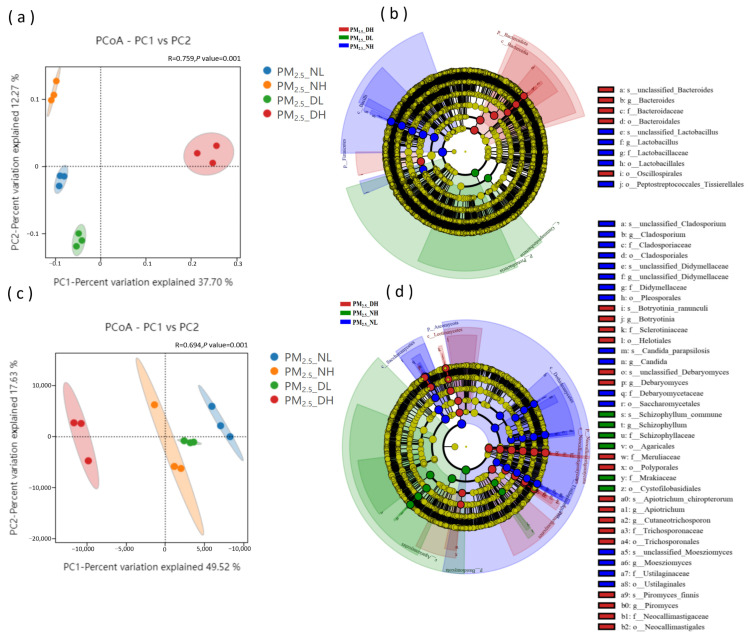
PCoA and LEfSe analysis of bacterial (**a**,**b**) and fungal (**c**,**d**) differences between PM_2.5_ samples.

Figure 2 shows that the confidence ellipses of bacteria (Figure 2a) and fungi (Figure 2c) in different groups are separated, indicating differences in the composition of bacteria and fungi in PM_2.5_ samples under the four groups. LEfSe analysis further identified the marker microorganisms in each group. *Bacteroides* and *Lactobacillus* were identified only at high and low altitude during the day, respectively. Their relative abundance in the corresponding group samples was significantly different from that of other groups, and their evolutionary relationship from order to species level bacteria can show differences (Figure 2b). Compared with bacteria, more marker fungi were identified (Figure 2d). The LDA value of *Piromyces* was the highest at high altitude during the day, followed by *Apiotrichum*, *Cutaneotrichosporon*, *Debaryomyces* and *Botryotinia*. *Moesziomyces*, *Candida* and *Cladosporium* were identified at low altitude at night, and *Schizophyllum* was identified at high altitude at night.

### 3.4. Identification of Harmful Microorganisms in PM_2.5_ and Network Interactions among Microorganisms

BugBase phenotype prediction identified 14 potential pathogenic bacterial genera in PM_2.5_ samples (Table 4), with a total relative abundance between 12.01% and 13.82%. The distribution of different bacterial genera in different types of PM_2.5_ was different. Among them, *Acinetobacter* had the greatest potential hazard and were the dominant bacteria in all samples (relative abundance >1%), while their relative abundance was not significantly different among various PM_2.5_ samples. The potential hazards of *Bacteroides* and *Staphylococcus* were slightly weaker than *Acinetobacter*, but they were also common opportunistic pathogens. *Bacteroides* was significantly higher in PM_2.5_ samples at high altitude during the day than in other categories, and *Staphylococcus* was significantly higher in PM_2.5_ samples at high altitude at night than in other categories (*p* < 0.05). In addition, referring to 123 fungal allergens published by Birgit et al. [19], 5 fungal allergens were found in PM_2.5_ samples (Table 5). *Aspergillus* was a potentially harmful bacterium, which was significantly lower than other environments at night, while the total abundance of potential allergens in this type of samples was the highest.

**Figure 3:**
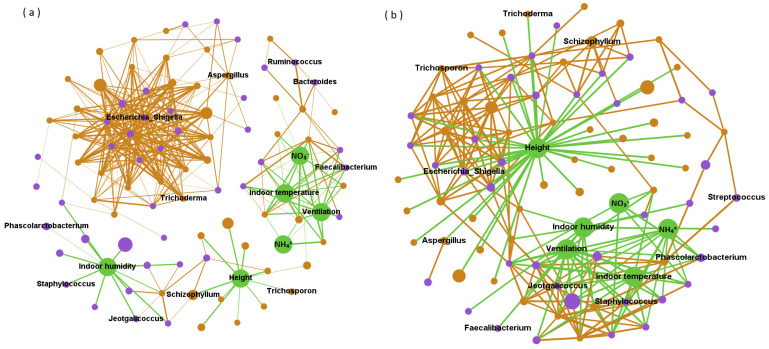
Network of positive (**a**) and negative (**b**) correlations among bacteria (purple), fungi (brown) and environmental parameters (green). Note: The figure shows the relative abundance of the top 50 bacteria and fungi, and the size of the circle corresponds to the level of their relative abundance. The potentially harmful microorganisms and environmental variables were labeled.

Network analysis revealed network interactions among bacteria, fungi, and environmental variables (Figure 3), and used the degree and betweenness centrality in the network structure to indicate the direct and indirect impacts on microbial abundance, respectively. Bacteria mainly played a positive role in the network structure. Among them, ten kinds of bacteria such as *Escherichia_Shigella*, *Prevotellaceae_NK3B31_group* and *Coprococcus* can directly had a positive impact on the abundance of more than sixteen microorganisms (degree ≥ 16). Environmental variables mainly indirectly positively affected microbial abundance. In this study, the betweenness centrality values of Indoor humidity, Height and NO_3_^−^ in the network structure were all higher than 1500 (with an average of 222). In terms of negative correlation, Height, Indoor temperature and Ventilationd were environmental parameters that directly negatively affected multiple microorganisms (the degrees were 38, 13 and 13, respectively). Fungi *Epicoccum*, *Debaryomyces* and *Piromyces* can also have a direct negative impact on a variety of microorganisms, and their degree values were 11. In addition, the indirect negative correlation between height and microbial abundance was stronger than other factors. The betweenness centrality value of height in the negative correlation network was 2081, while that of Campylobacter was only 620.

### 3.5. Traceability Analysis

**Figure 4:**
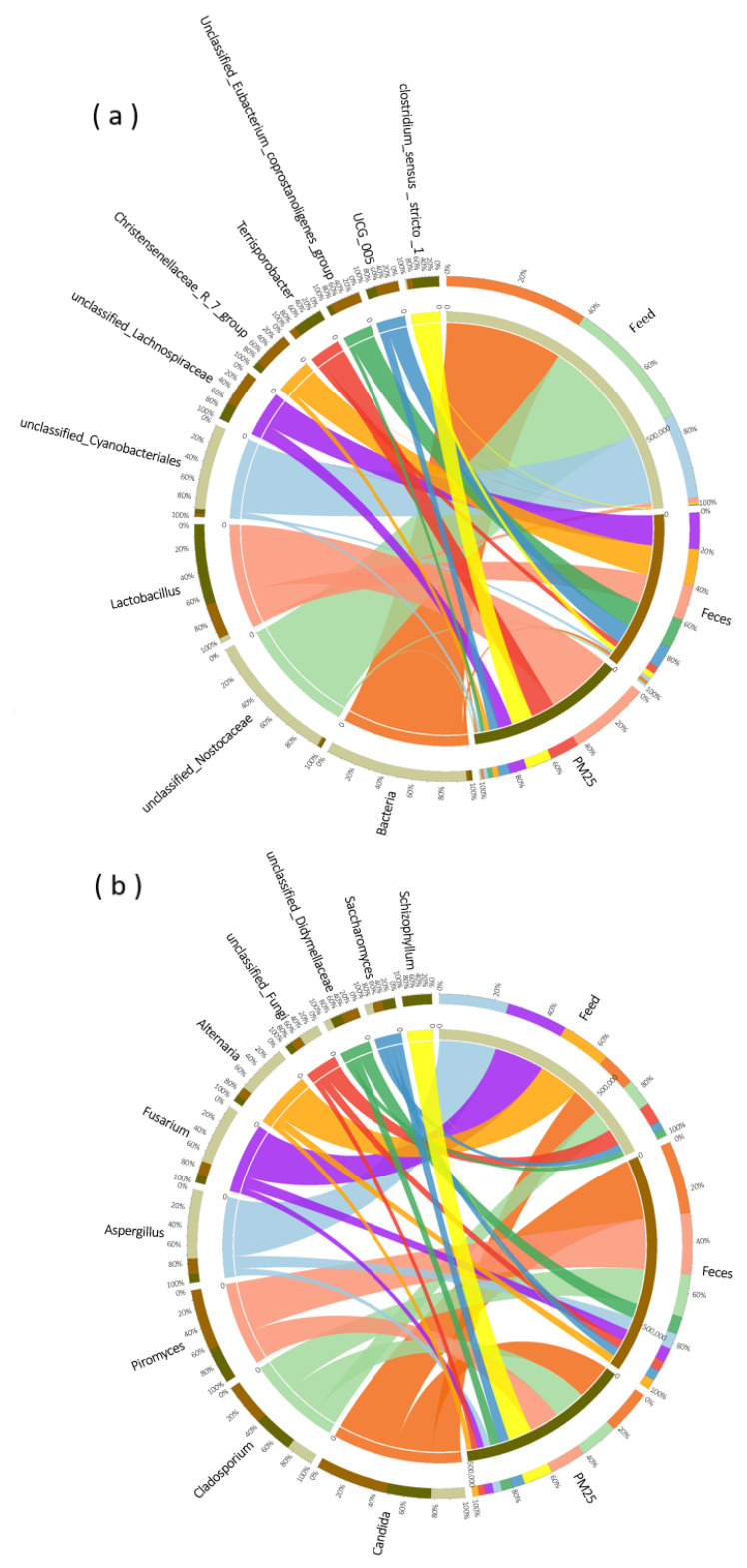
Species composition of bacteria (**a**) and fungi (**b**) in PM_2.5_, feed and feces samples.

The composition of the top ten dominant bacteria and fungi in feed, feces and PM_2.5_ samples were compared (Figure 4). In both in PM_2.5_ and feces, *Lactobacillus*, *Terrisporobacter*, *Clostridium_sensu_stricto_1*, *unclassified_Lachnospiraceae* and *UCG_005* were the main dominant bacteria. There was only a small amount of *Lactobacillus* and *Clostridium_sensu_stricto_1* in feed, with the majority being *unclassified _ Nostocaceae* and *unclassified_Cyanobacteriales*. In terms of dominant fungi, only the relative abundance of *Schizophyllum* differed significantly between the PM_2.5_ and feces samples, with the other dominant fungi being close in species and abundance. The dominant fungi in feed were also present in PM_2.5_, although there was a large difference in relative abundance.

**Figure 5:**
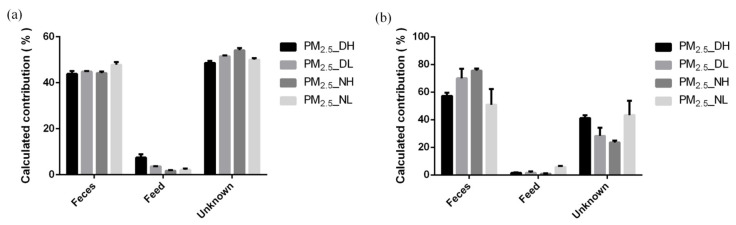
Traceability analysis of bacteria (**a**) and fungi (**b**) by SourceTracker method.

SourceTracker calculation results showed that manure in the pig house was the primary source of airborne microorganisms, while feed contributed little (Figure 5). In this experiment, the contribution of feces to airborne bacteria ranged from 42.77% to 49.07%, while the contribution of feed was only 1.44–8.69%. Moreover, the contribution of feces to airborne fungi was from 43.53–75.23%, and the contribution of feed was 0.59–6.40%. After five SourceTracker runs, it was observed that the fecal source and the feed source of PM_2.5_ samples, both collected at 1.7 m at night, had a lower RSD value and a higher RSD value, respectively (Table 6).

## 4. Discussion

### 4.1. Environmental Variables inside the Pig House

The PM_2.5_ concentrations in this experiment were close to those reported in previous studies [20] and exceeded the limit of 35 μg·m^−3^ in the Ambient Air Quality Standard (GB3095-2012) issued by the Ministry of Environmental Protection in China. During the spring season, because the pig house had a low density of pigs and a ventilation level ranging from 6.4 to 47.0 m^3^·h^−1^, PM_2.5_ concentrations close to 200 were not reached, as reported by Shang et al. [21]. However, it should be noted that the latest PM_2.5_ limit proposed by the World Health Organization (WHO) has been lowered from 25 to 10 μg·m^−3^, indicating that even low concentrations of PM_2.5_ can be hazardous to humans and animals.

The environmental variables were higher during the day than at night, which may be related to the higher temperature and the more frequent activity of pigs during the day than at night. Some studies have found that PM concentrations in pig houses are significantly affected by pig activity [22,23]. When the pigs were fed or frightened, their activity increased and PM concentration increased significantly. Additionally, as this experiment was carried out in spring, the diurnal temperature changes outside the house had a direct impact on indoor temperature and microbial activity. Higher temperatures led to an increase in microbial activity and pollutant emissions, such as ammonia, and negatively affected air quality [24]. In addition, during the monitoring period, the concentration of PM_2.5_ at high altitude (1.7 m) were significantly higher than that at low altitude (0.6 m). This may be attributed to the light mass of PM_2.5_, which is more easily suspended in the air than coarse particles. Understanding the diurnal changes of the environmental parameters, such as PM_2.5_, in the pig house can help regulate the environment in the pig house more precisely.

Inorganic secondary ions, represented by NH_4_^+^ and NO_3_^−^, can be used as marker substances for identifying the production of secondary particles from air pollutants such as ammonia (NH_3_) and nitrogen oxides (NO_x_) in the house. This process takes place through a series of chemical reactions between acidic and alkaline gases as precursors, and has been detected in the air environment in several scenarios in recent years [25,26]. The content of NH_4_^+^ and NO_3_^−^ detected during the day were significantly higher than that at night (*p* < 0.05), which was related to increased pig activity during the day and poorer air quality in the house. Although Roumeliotis et al. [27] found that more than 50% of PM_2.5_ in European chicken houses was derived from secondary particles, the proportion of water-soluble ions in PM_2.5_ in the pig house in this study was very low. This suggests that the PM_2.5_ in this environment was predominantly produced directly by some substances in the house.

### 4.2. Airborne Microbial Varies and Network Interaction

The α diversity showed that compared with airborne fungi, the airborne bacteria in the pig house seemed to be more abundant and have a more complex community structure. Yan et al. found that bacteria accounted for more than 90% of PM_2.5_ microbial components in pig houses using metagenomic technology, which was consistent with the findings of this study [28]. Eisenlöffel et al. [29] measured the concentration of airborne bacteria in a pig house to be 2.2–5.2 × 10^5^ colony-forming units (CFU)/m^3^. In the composition of of phylum level airborne microorganisms, *Firmicutes* was the most dominant bacterial phylum, which was consistent with previous research conducted in piggeries [30,31]. Conversely, some studies have reported that the most abundant phylum in pig farms bioaerosols is *Proteobacteria* rather than *Firmicutes* [32]. This discrepancy may be caused by the collection of samples from the external environment surrounding the piggery, because Du et al. [33] believed that *Proteobacteria* was the most abundant bacterial phylum in residential areas, and its proportion was greatly different from that of the PM_2.5_ in piggeries. Therefore, the composition of airborne microorganisms in pig houses may be affected by the point locations where the samples are collected. The point locations outside the pig house may vary in many ways depending on the external environment, whereas the airborne microbial community structure inside the pig house is relatively stable.

*Lactobacillus* and *Acinetobacter* are the most noteworthy bacteria in the generic microbial composition. *Lactobacillus* has the highest abundance among the identified bacteria, and is commonly found in nature. It rarely causes diseases and plays an important role in intestinal health of pigs. Branched chain fatty acids (BCFAs) produced by *Atopostipes* have an important influence on dietary digestion [34]. In contrast, *Acinetobacter* is the most harmful to health among the identified potential pathogens. Tang et al. [35] identified *Acinetobacter*, *Streptococcus*, *Escherichia-Shigella*, and *Pseudomonas* as the pathogenic bacteria, in both PM_2.5_ and the respiratory tract of pigs. *Acinetobacter* can easily cause respiratory tract infection, bacteremia, meningitis, urogenital tract wound and skin infection while the body’s resistance weakens or skin damage occurs [36]. *Acinetobacter* has strong adhesion ability and grows easily in humid environments. It has been found many times in breeding environments in the past reports [37]. As far as fungi are concerned, there are many kinds of *Candida*, but only a few of them can cause diseases to humans, and *Candida albicans* is the most common one. Attention should be paid to the aflatoxins produced by Aspergillus flavus among potential sensitizing fungi; they have been classified as Class 1 carcinogens by the Cancer Research Institute of the WHO, and can damage human and animal liver tissues. In severe cases, they can lead to liver cancer and even death.

Due to the diurnal variation and height, the microbial composition of the four types of PM_2.5_ samples showed significant differences (Adonis; *p* < 0.01). However, the specific effects of altitude, daytime and night on airborne microorganisms remain unclear, possibly due to the complex effects of environment on microbial communities. Network analysis has revealed that certain indicators such as temperature and altitude are positively correlated with some factors, but negatively correlated with others. Combinations such as intersymbiosis, co-colonization and niche overlap make bacteria and fungi coexist under various interactions [38,39]. In this study, we found a positive correlation between bacteria and microbial community, which was consistent with the results reported by Ma et al. [5] Microorganisms in PM_2.5_ have strong coagulation and modularization characteristics. Compared with fungi, bacteria have more complex interactions, especially in positive correlation networks. There are 10 kinds of bacteria that can directly affect other microorganisms to a higher degree, while 3 kinds of fungi can negatively affect many kinds of microorganisms directly. As for environmental factors, they mainly indirectly affect microbial abundance. Fungal spores in the air will be affected by short-term temperature changes, vertical heat flow and mechanical agitation [40]. In addition, a humid environment is more conducive to microbial growth, and NO^3−^ is considered as a nutrient for bacterial growth and metabolic activities, so they are mainly positively correlated. Airborne microorganisms in piggeries have temporal and spatial variations, but environmental effects may differ across different genera. The interaction of bacteria in airborne microorganisms is mainly synergistic.

### 4.3. Traceability Analysis

In this study, the microbial compositions of these three groups were compared. The compositions of dominant bacteria and fungi in feed and PM_2.5_ were very different, contradicting the previous view that feed is the main source of PM_2.5_ in piggeries [41]. The microbial composition of PM_2.5_ was consistent with that reported previously, and most of the microbial composition in feed was from animals, while most of the microbial composition in feed was from plants. The possible reason may be the formula feed is widely used in pig feed now, which is mainly composed of energy feed, protein feed and premix. The energy feed and protein feed as the main body of the formula mostly use plant raw materials such as corn, soybean meal, and rapeseed meal. Secondly, the nutritional components of feed itself, the production process, the content of additives and other aspects may also affect its microbial composition to a certain extent [42]. The bacterial composition of feces was similar to that of PM_2.5_ and both of them had the highest relative abundance of *Lactobacillus*, a kind of bacterium which can colonize the intestinal tract of pigs [43]. Thus, the relative abundance of *Lactobacillus* in feces increase continuously, which may be the main reason why *Lactobacillus* in feces was much higher than that in feed. Moreover, except *Schizophyllum*, other fungi in feces were similar to PM_2.5_ samples. To sum up, the microbial community structure of PM_2.5_ and feces were similar, while the feed seemed to be very different.

Further calculations by SourceTracker showed that the fecal source of PM_2.5_ in the air of the closed pig house was much more than feed. SourceTracker works by modeling environment samples as mixed sinks of several sources. It then assigns all the OTUs in the environmental sink sample to one source and classifies an OTU as “Unknown” if it cannot be assigned to a source. In this study, the unknown source of the PM_2.5_ microbial community accounted for a large proportion, and the taxa of the unknown source may come from unidentified sources in the piggery, such as external air, soil, pig dander and hair, etc. [44], and may also include the air protozoan microbial community in the piggery. The SourceTracker method has limitations in identifying the sources with similar bacterial communities [45] and needs to be further optimized in its running speed and result accuracy. To enhance the reliability of SourceTracker’s proportional prediction, RSD was calculated by 5 independent SourceTracker runs. The source with the largest proportion and the smallest RSD value indicates higher confidence, such as the fecal source of PM_2.5_ samples with a height of 1.7 m collected at night. For the feed source for this sample, the program shows greater variability in quantifying low source contributors. SourceTracker may only predict the presence of a low proportion of sources and cannot quantify a low proportion of source inputs. RSD values of all predicted sources in all PM_2.5_ samples were less than 100%, indicating consistent results when applying the SourceTracker setting of this experiment.

## 5. Conclusions

There are abundant potential pathogenic bacteria on PM_2.5_ in piggeries. In this study, DNA was extracted from PM_2.5,_ feces and feed samples in the piggery and 16S high-throughput sequencing was performed. The results indicate that the composition and abundance of the bacterial community between samples were significantly higher than those of fungi. We identified 14 potential pathogens and 5 allergens in PM_2.5_. *Acinetobacter* with relative abundance >1% in all samples was the most potentially harmful, followed by *Bacteroides* and *Staphylococcus*. The network analysis revealed that bacteria played a positive role in the network structure, and environmental variables mainly indirectly and positively affected microbial abundance On this basis, the SourceTracker program was used to trace the source of PM_2.5_ samples, and it was found that the contribution of feces was significantly higher than that of feed. In conclusion, this study provides important evidence that PM_2.5_ airborne microorganisms in piggeries mainly come from piggery feces. Future research should investigate the relationship between airborne microorganisms and feces in pig houses and explore the regulation of harmful microorganisms in the air of livestock houses by taking measures to treat feces.

## Figures and Tables

**Table 1:** Sample information and group name.

Sample Information		Group Name
Samples collected during the day	High altitude	PM_2.5__DH
	Low altitude	PM_2.5__DL
Samples collected at night	High altitude	PM_2.5__NH
	Low altitude	PM_2.5__NL
Feed samples during sampling		Feed
Fecal samples during sampling		Fece

**Table 2:** Environment variables inside the pig house during the experiment.

Item		PM_2.5_ (μg·m^−3^)	Ventilation (m³·h^−1^)	Humidity (%)	Temperature (°C)	NH_4_^+^ (μg∙m^−3^)	NO_3_^−^ (μg∙m^−3^)
High altitude during the day (1.7 m; 7:30–19:30)	Mean ± SD	64.47 ± 19.07 ^a^	19.26 ± 18.36 ^a^	59.1 ± 14.51 ^a^	20.37 ± 4.36 ^a^	3.67 ± 0.12 ^a^	1.17 ± 0.09 ^a^
	Max	95.61	47	78.76	25.12	3.92	1.31
	Min	40.09	6.4	41.03	16.01	3.57	1.08
Low altitude during the day (0.6 m; 7:30–19:30)	Mean ± SD	55.25 ± 17.46 ^b^	19.26 ± 18.36 ^a^	61.09 ± 12.29 ^a^	20.64 ± 4.18 ^a^	4.57 ± 0.41 ^b^	1.21 ± 0.06 ^a^
	Max	90.32	47	79.11	25.18	5.14	1.29
	Min	37.91	6.4	40.06	16.63	4.12	1.12
High altitude at night (1.7 m; 19:30–7:30)	Mean ± SD	46.47 ± 15.63 ^b^	7.18 ± 1.33 ^b^	74.27 ± 10.01 ^b^	15.19 ± 2.41 ^b^	2.97 ± 0.21 ^c^	0.77 ± 0.03 ^b^
	Max	76.85	10	88.43	18.12	3.39	0.81
	Min	30.34	6.4	55.12	12.31	2.73	0.73
Low altitude at night (0.6 m; 19:30–7:30)	Mean ± SD	41.25 ± 12.62 ^c^	7.18 ± 1.33 ^b^	75.31 ± 8.82 ^b^	15.89 ± 3.15 ^b^	2.2 ± 0.13 ^c^	0.67 ± 0.05 ^b^
	Max	60.61	10	87.17	18.92	2.41	0.72
	Min	22.91	6.4	56.31	12.71	2.07	0.61

Note: Different superscript letters in the same column represent significant variation (*p* ≤ 0.05), while the same letter represents no significant variation (*p* > 0.05). SD, standard deviation.

**Table 3:** α diversity index of different groups of samples.

The α Diversity Index	Sample	Bacterial	Fungi
ACE	PM_2.5__DH	1481.53 ± 44.1 ^a^	511.79 ± 44.12 ^b^
	PM_2.5__DL	1064.23 ± 47.13 ^a^	458.72 ± 17.72 ^b^
	PM_2.5__NH	911.16 ± 21.78 ^a^	324.83 ± 14.94 ^b^
	PM_2.5__NL	1239.42 ± 44.33 ^a^	411.39 ± 90.88 ^b^
	Feces	935.49 ± 48.66 ^a^	410.13 ± 20.92 ^b^
	Feed	889.8 ± 12.54 ^a^	472.49 ± 6.06 ^b^
Shannon	PM_2.5__DH	8.42 ± 0.06 ^a^	6.15 ± 0.07 ^b^
	PM_2.5__DL	7.97 ± 0.04 ^a^	6.48 ± 0.13 ^b^
	PM_2.5__NH	8.14 ± 0.04 ^a^	5.9 ± 0.04 ^b^
	PM_2.5__NL	6.95 ± 0.14 ^a^	6 ± 0.13 ^b^
	Feces	7.83 ± 0.02 ^a^	6.36 ± 0.12 ^b^
	Feed	7.56 ± 0.03 ^a^	6.35 ± 0.03 ^b^

The values are shown as mean ± standard deviation (SD). Means within each row followed by different superscript letters were significantly different (*p* < 0.05).

**Table 4:** The relative abundance of potential pathogenic bacteria in PM_2.5_ samples.

Bacteria	PM_2.5__DH	PM_2.5__DL	PM_2.5__NH	PM_2.5__NL
Phascolarctobacterium	2.73 ± 0.05	2.59 ± 0.02	2.76 ± 0.03	3.14 ± 0.1
Streptococcus	1.59 ± 0.06	1.92 ± 0.07	2.09 ± 0.05	1.82 ± 0.11
Bacteroides	2.65 ± 0.18	0.47 ± 0.03	0.37 ± 0.01	0.51 ± 0.01
Acinetobacter	1.14 ± 0.04	1.31 ± 0.02	1.29 ± 0.04	1.07 ± 0.03
Ruminococcus	1.1 ± 0.03	0.73 ± 0.04	0.7 ± 0.02	0.86 ± 0.02
Prevotella	0.9 ± 0.18	1.25 ± 0.09	1.32 ± 0.02	1.22 ± 0.1
Escherichia_Shigella	0.79 ± 0.04	1.51 ± 0.01	0.93 ± 0.02	1.61 ± 0.01
Staphylococcus	0.37 ± 0.01	0.45 ± 0.06	1.17 ± 0.05	0.73 ± 0.11
Faecalibacterium	0.86 ± 0.05	0.59 ± 0.04	0.43 ± 0.02	0.53 ± 0.02
Jeotgalicoccus	0.24 ± 0.02	0.32 ± 0.01	0.69 ± 0.04	0.44 ± 0.02
Treponema	0.23 ± 0.02	0.31 ± 0.02	0.17 ± 0.01	0.25 ± 0.01
Pseudomonas	0.22 ± 0.01	0.24 ± 0.02	0.15 ± 0.02	0.13 ± 0.01
Oscillospira	0.17 ± 0.02	0.29 ± 0.03	0.29 ± 0.03	0.32 ± 0.08
Achromobacter	0	1.04 ± 0.04	0	0.03 ± 0.01
Total bacterial pathogen	13 ± 0.69	13.03 ± 0.43	12.36 ± 0.34	12.66 ± 0.45

**Table 5:** The relative abundance of potential allergens in PM_2.5_ samples.

Fungi	PM_2.5__NH	PM_2.5__DH	PM_2.5__NL	PM_2.5__DL
Schizophyllum	13.27 ± 0.42	7.33 ± 0.23	4.43 ± 0.07	4.23 ± 0.21
Aspergillus	1.71 ± 0.13	2.13 ± 0.19	2.27 ± 0.12	2.32 ± 0.02
Trichosporon	1.29 ± 0.11	1.59 ± 0.1	1.16 ± 0.22	1.07 ± 0.08
Trichoderma	0.57 ± 0.04	0.01 ± 0.01	0.71 ± 0.11	0.62 ± 0.09
Wallemia	0.04 ± 0.01	0.09 ± 0.02	0.04 ± 0.01	0.06 ± 0.02
Total fungal allergen	16.87 ± 0.71	11.16 ± 0.56	8.61 ± 0.53	8.29 ± 0.42

**Table 6:** Relative Standard Deviation Analysis of SourceTracker Results after Five Independent Runs of Select Samples.

Sample	Source	Bacteria		Fungi	
		Average Proportion (%)	RSD	Average Proportion (%)	RSD
PM_2.5__DH	Feces	43.5%	1.59%	57.4%	0.32%
	Feed	7.5%	1.52%	1.6%	4.62%
	Unknown	49.0%	1.20%	41.1%	0.40%
PM_2.5__DL	Feces	45.5%	1.15%	69.6%	0.75%
	Feed	3.6%	3.86%	1.5%	7.42%
	Unknown	50.9%	0.98%	28.9%	1.71%
PM_2.5__NH	Feces	44.1%	0.92%	75.5%	0.19%
	Feed	1.8%	5.10%	1.1%	10.48%
	Unknown	54.2%	0.62%	23.4%	0.55%
PM_2.5__NL	Feces	48.4%	1.26%	50.5%	1.74%
	Feed	2.2%	3.11%	5.9%	4.67%
	Unknown	49.4%	1.25%	43.6%	1.44%

## Data Availability

The data that support the findings of this study are available from the corresponding author, upon reasonable request.
